# Association of adiponectin with cognitive function precedes overt diabetes in the Brazilian Longitudinal Study of Adult Health: ELSA

**DOI:** 10.1186/s13098-018-0354-1

**Published:** 2018-07-09

**Authors:** Adriana Cezaretto, Claudia Kimie Suemoto, Isabela Bensenor, Paulo A. Lotufo, Bianca de Almeida-Pititto, Sandra R. G. Ferreira, the ELSA Research Group, Estela M. L. Aquino, Eduardo L. A. Mota, Sandhi Maria Barreto, Valéria Maria Azeredo Passos, Dóra Chor, Marilia S. Carvalho, Isabela M. Bensenor, Paulo A. Lotufo, José Geraldo Mill, Maria Del Carmen Molina, Bruce B. Duncan, Maria Inês Schmidt, Moyses Szklo

**Affiliations:** 1 Department of Epidemiology, School of Public Health, University of Sao Paulo, São Paulo, Brazil; 2 Division of Geriatrics, Medical School, University of Sao Paulo, São Paulo, Brazil; 3 Department of Internal Medicine, Medical School, University of Sao Paulo, São Paulo, Brazil

**Keywords:** Adiponectin, Prediabetes, Cognitive function, Dementia, Prevention

## Abstract

**Background:**

Adiponectin is an insulin-sensitizer adipocytokine endowed with neuroprotective actions. Whether adiponectin regulates neuronal functioning toward delaying cognitive decline independently of the glucose metabolism disturbance has been poorly explored. This study evaluated if the performance in cognitive tests was associated with adiponectin levels prior the development of type 2 diabetes in middle-aged individuals.

**Methods:**

A sample of 938 non-diabetic participants of ELSA had their cognitive function assessed by the CERAD delayed word recall test, the verbal fluency test and the trail making test. Stepwise multiple linear regression using forward selection had the response to cognitive tests as the dependent variable and adiponectin as the independent variable of main interest, adjusted for glucose tolerance status and confounders.

**Results:**

Mean age was 45.7 ± 4.9 years, 54.5% were women, 43.0% had high education level, 59.3% weight excess and 70.0% prediabetes. In crude model, only the delayed recall memory was associated with adiponectin levels. In an initial regression model, delayed recall memory remained independently associated with adiponectin levels and prediabetes. After complete adjustments, adiponectin but not prediabetes maintained independently associated with delayed recall memory (β 0.067; 95% CI 0.006–0.234; p = 0.040). On the other hand, learning memory showed to be associated with prediabetes (β 0.71 95% CI 0.17; 1.24; p = 0.009) but not with adiponectin.

**Conclusions:**

The association of memory with adiponectin in middle-aged individuals, prior overt diabetes, suggests that this adipocytokine could anticipate cognitive impairmentρ detection, when preventive strategies could be more effectively implemented. The usefulness of adiponectin to identify increased risk for cognitive dysfunction before advanced age needs to be prospectively investigated in ELSA cohort.

## Background

The importance of adiponectin discovery was initially based on its role on the glucose metabolism and obesity-related diseases [1, 2]. This adipocytokine exerts insulin-sensitizing effects via specific receptors (AdipoRs) expressed in several tissues [3]. Benefits of adiponectin on immune system and neuronal function were further reported [4]. In the brain, AdipoRs were found in hippocampus, cortex and hypothalamus, and adiponectin administration to animals induced neurogenesis [5]. Its anti-inflammatory and neuroprotective actions motivated investigations on the pathophysiology of neurodegenerative disorders such as dementias [6, 7].

Prevalence of dementias is high in elderly living in developing an developed countries; Alzheimer’s disease is the most common cause of dementia in older adults and the sixth leading cause of death in the United States [8, 9].

A metanalysis revealed that type 2 diabetes mellitus (DM) was associated with 39% increased risk of Alzheimer’s disease, and 138% increased risk of vascular dementia compared to non diabetic individuals [10]. The combination of these diseases potentiates morbidity and accelerates mortality mainly from cardiovascular events [10, 11]. Several lines of evidence have shown that neuroinflammation and insulin resistance play an important role in the pathogenesis of dementia [12], explaining its association with metabolic disorders such as DM. Since hippocampal cognitive functioning depends on the insulin-stimulated glucose uptake, states of insulin resistance might contribute to impair memory performance [13, 14]. Extracellular amiloid-β accumulation, synaptic loss and neuronal death are some pathophysiological features attributed to insulin resistance [12, 15]. Even in prediabetic phase, there is some evidence of a reduced cognitive function [16]. Since insulin has a central role in learning and memory, and adiponectin an insulin-sensitizing effect, the latter represents a potential therapeutic target against dementia.

Considering that neuropathological lesions start 20 years before the onset of clinically symptoms [17], early detection of cognitive decline might be useful to prevent dementia through lifestyle changes and pharmacological interventions [18]. Whether adiponectin exerts protective effect on glucose metabolism and neuronal functioning delaying the onset of metabolic and neurodegenerative diseases in middle-aged individuals remains poorly investigated.

The Longitudinal Study of Adult Health (ELSA-Brasil) is multicenter study designed to investigate the incidence of DM and cardiovascular disease, and their biological, behavioral, environmental, occupational, psychological and social risk factors. This also represents a unique opportunity to investigate association of an early marker of dementia risk. We hypothesized that adiponectin concentration could be associated with improved cognitive performance before advanced age, independently of the glucose metabolism status. We aimed to evaluate if the performance in cognitive tests was associated with adiponectin levels, in middle-aged non-diabetic participants at the baseline of the ELSA-Brasil.

## Methods

### Participants

Objectives, study population, and methods of the ELSA-Brasil were previously reported [19, 20]. Briefly, 15,105 (54% women) active or retired employees of six Brazilian universities, aged 35–74 years, were eligible for baseline examinations of the cohort study. They had an initial interview and then scheduled for clinical examination and laboratory tests in research centers. This study was approved by the institutional ethics committee of the University of Sao Paulo, and written consent was obtained from all participants.

The present cross-sectional analysis was performed with baseline data of a sample of 1000 individuals from the 5061 participants of the research center in São Paulo. Inclusion criteria were 35–54 years of age; exclusion criteria were clinical history of DM and/or cardiovascular disease; use of medications which could interfere in the cognitive function (neuroleptics, antiparkinsonian and anticonvulsant agents); C-reactive protein concentration > 10 mg/L. Sixty participants did not meet eligibility criteria, and two individuals were excluded from analyses due to insufficient aliquot volume for biomarkers determinations [21]. Participants were interviewed using standardized questionnaires to obtain sociodemographic and lifestyle data. Afterwards, they were scheduled for clinical examination and laboratory procedures.

### Cognitive function assessment

The neuropsychological tests used for the assessment of cognitive function were: the Consortium to Establish a Registry for Alzheimer’s Disease (CERAD) delayed word recall test [22], fluence test and trail making test, version B. A Brazilian version of CERAD including a ten-word list was used to evaluate verbal learning memory, delayed recall memory, and recognition of verbal memory [23, 24]. Participants were asked to read 10 words and, after 5 min, they had 60 s to record these words. The score was equal to the number of recalled words. The semantic (animal category) and phonemic (letter F) verbal fluency tests were used to evaluate the language domain, and the score corresponded to the total number of generated words by the participant [24]. The higher the scores the better cognitive function in those domains. The trail making test was used to evaluate executive function, as it is related to attention, concentration, and psychomotor speed. The score corresponded to the time (in seconds) taken to complete the test. The longer the time taken to complete, the worse the participant’s performance.

### Other measures

The presence of depression was assessed by the Clinical Interview Schedule—Revised [25] and diagnosed according to ICD-10 [26]. Physical activity level was assessed using the International Physical Activity Questionnaire, and took account of the sum of the activities related to leisure and commuting [27, 28]. Body weight and height were measured using calibrated electronic scales and a fixed rigid stadiometer. Body mass index (BMI) was calculated as weight (kilograms) divided by squared height (meters). Abdominal circumference was measured with an inextensible tape. Blood pressure was taken three times after a 5-min rest in the sitting position; the mean of the second and third measurements was used [29]. Hypertension was defined as systolic or diastolic blood pressure higher than 140/90 mmHg or antihypertensive treatment.

Participants underwent a 2-h 75-gram oral glucose tolerance test. Prediabetes was defined according to American Diabetes Association criteria [30]. Insulin resistance was assessed by the homeostasis model assessment-insulin resistance (HOMA-IR) index. Dyslipidemia was characterized by LDL-cholesterol ≥ 130 mg/dL and/or lipid-lowering agents use. Hypothyroidism was defined by TSH levels > 4.0 IU/L and free T4 < 0.8 µg/dL, while hyperthyroidism by THS levels < 0.4 IU/L and free T4 ≥ 1.9 µg/dL.

### Analytical procedures

Plasma glucose was determined by the hexokinase method, and HDL-cholesterol by homogeneous colorimetric, without precipitation (ADVIA Chemistry; Siemens, Deerfield, Illinois, USA). LDL-cholesterol was calculated using the Friedewald equation. When triglyceride concentration was greater than 400 mg/dL, the LDL-cholesterol level was directly measured [31]. Immunochemistry was used to measure high-sensitivity C-reactive protein (Dade Behring, Siemens, Marburg, Germany), and enzyme-linked immunoenzymatic assay for insulin (Siemens, Tarrytown, USA) and adiponectin determinations (Enzo Life Sciences, Farmingdale, NY, USA). Intra-assay and inter-assay coefficients of variation ranged from 1.8 to 7.2%, and from 0.9 to 14.4%, respectively [21].

### Statistical analysis

Descriptive data were shown as frequencies of categorical variables, or as mean and standard deviation (SD) for continuous variables. Student *t* test or Chi square test was used to compare variables between normotolerant with prediabetic participants. Continuous variables with skewed distribution were log-transformed (adiponectin, HOMA-IR and C-reactive protein) or Mann–Whitney test was used. Simple linear regression was used to test association of cognitive function domains with clinical variables and adiponectin. Cognition domains (learning memory, delayed recall memory, recognition memory, trail test, and fluency tests) were the dependent variables, adiponectin the independent variable of main interest in inward multiple linear regression analyses. Due to the recognized association of glucose metabolism disturbance with cognition performance, association of adiponectin with cognitive function was initially tested in the presence of prediabetes. Afterwards, models were adjusted for demographics, anthropometric data, lifestyle variables (physical activity, smoking and alcohol use), HOMA-IR, C-reactive protein, hypertension, dyslipidemia, depression and thyroid dysfunction. Additionally, an interaction term (adiponectin*HOMA-IR) was tested. SPSS software, version 19.0 for Windows (SPSS Inc., Chicago, Illinois, USA) was used. A p-value of 0.05 was considered significant.

## Results

The mean age of 938 participants was 45.7 years (SD 4.9); 54.5% were women and nearly 70% had prediabetes. A higher frequency of women had university level compared to men (52.3% versus 30.9%; p < 0.001). Moreover, women were less likely to consume alcohol (67.9% versus 76.3%, p < 0.001) and to be current smokers (14.7% versus 22.5%, p < 0.001) than men. Lower abdominal circumference (83.3 ± 10.7 versus 91.3 ± 10.7 kg/m^2^, p = 0.031), systolic and blood pressure levels (112 ± 13 versus 122 ± 14 and 72 ± 10 versus 78 ± 10 mmHg, p < 0.001), fasting plasma glucose (100.0 ± 7.3 versus 105.0 ± 8.7 mg/dL, p < 0.001), and higher adiponectin concentration (13.8 ± 18.6 versus 12.3 ± 15.1 µg/mL, p < 0.001) and better performance in domains of cognitive function than men (data not shown in tables).

Stratifying by the glucose tolerance status (Table 1), more men than women had prediabetes. Prediabetic individuals were older, had higher mean values of anthropometric data, blood pressure and biochemical variables compared to normotolerant ones. Furthermore, prediabetic individuals had lower adiponectin concentration and poorer scores of learning and delayed recall memories.

**Table 1:** Demographic, lifestyle, clinical, and cognitive domains data of participants stratifyed according to glucose tolerance status

	Normoglycemia N = 278	Prediabetes N = 660	p-value
Clinical variables			
Women (%)	71.9	47.1	< 0.001
Caucasian (%)	62.6	59.2	0.344
Current smoker (%)	18.0 (50)	18.3 (121)	0.071
Current alcohol user (%)	70.1 (195)	72.4 (478)	0.227
Physical activity (days/week)	1.30 (0.62)	1.31 (0.63)	0.846
Age (years)	44.9 (4.9)	46.1 (4.9)	< 0.001
Body mass index (kg/m^2^)	25.4 (3.7)	26.7 (4.1)	< 0.001
Abdominal circumference (cm)	82.5 (9.8)	88.4 (11.3)	< 0.001
Systolic blood pressure (mmHg)	112 (13)	119 (14)	< 0.001
Diastolic blood pressure (mmHg)	71 (10)	76 (10)	< 0.001
Plasma glucose (mg/dL)	94.4 (3.8)	105.7 (6.8)	< 0.001
Triglycerides (mg/dL)	101.3 (50.5)	138.6 (83.3)	< 0.001
LDL-cholesterol (mg/dL)	122.7 (28.7)	131.5 (33.5)	< 0.001
HDL–cholesterol (mg/dL)	59.3 (13.1)	53.7 (12.9)	< 0.001
HOMA-IR^a^	1.32 (1.01)	2.04 (1.58)	< 0.001
C-reactive protein^a^ (mg/L)	2.03 (2.13)	2.06 (2.01)	0.308
Adiponectin^a^ (μg/mL)	14.3 (23.5)	12.6 (13.5)	0.127
Cognitive function			
Learning memory	21.0 (3.6)	20.4 (3.9)	0.031
Delayed recall memory	7.1 (1.9)	6.7 (1.9)	0.015
Recognition memory	9.7 (0.7)	9.5 (0.9)	0.095
Semantic verbal fluency test	18.3 (4.7)	18.4 (4.8)	0.663
Phonemic verbal fluency test	12.3 (4.3)	12.4 (4.5)	0.727
Trail test	100.5 (65.9)	109.7 (70.3)	0.055

Data are presented by frequencies or means and standard deviations

^a^Mann–Whitney test used

Associations between domains of cognitive function and clinical variables are depicted in Table 2. In general, the domains of learning and delayed recall memories were the most significantly associated with clinical variables. Age, BMI, blood pressure levels, plasma glucose, and triglycerides were inversely associated with learning and delayed recall memories and directly with trail test scores. Some significant associations of insulin resistance index and C-reactive protein with selected domains of cognitive were also detected.

**Table 2:** Coefficients of simple linear regression between domains of cognitive function and clinical and biochemical data

	Learning memory		Delayed recall memory		Recognition memory		Semantic verbal fluency test		Phonemic verbal fluency test		Trail test	
	β	p-value	Β	p-value	Β	p-value	Β	p-value	β	p-value	Β	p-value
Age	− 0.105	0.001	− 0.142	< 0.001	0.004	0.896	− 0.081	0.013	− 0.041	0.208	0.259	< 0.001
Body mass index	− 0.105	0.001	− 0.089	0.006	− 0.038	0.253	− 0.062	0.057	− 0.046	0.162	0.092	0.005
Systolic BP	− 0.124	< 0.001	− 0.154	< 0.001	− 0.082	0.012	− 0.111	0.001	− 0.119	< 0.001	0.117	< 0.001
Diastolic BP	− 0.082	0.013	− 0.119	< 0.001	− 0.073	0.026	− 0.109	0.001	− 0.125	< 0.001	0.101	0.002
Plasma glucose	− 0.108	< 0.001	− 0.127	< 0.001	− 0.035	0.287	− 0.016	0.634	− 0.058	0.079	0.095	0.004
Triglycerides	− 0.138	< 0.001	− 0.173	< 0.001	− 0.068	0.040	− 0.065	0.050	− 0.044	0.183	0.075	0.024
HDL-cholesterol	0.135	< 0.001	0.187	< 0.001	0.070	0.034	0.092	0.005	0.093	0.005	− 0.093	0.005
LDL-cholesterol	0.037	0.261	0.030	0.359	− 0.002	0.947	0.025	0.450	0.023	0.484	− 0.039	0.242
HOMA-IR^a^	− 0.087	0.008	− 0.089	0.007	− 0.012	0.722	− 0.015	0.653	− 0.043	0.192	0.004	0.911
CRP^a^	− 0.032	0.329	− 0.056	0.085	− 0.019	0.554	− 0.105	0.001	− 0.046	0.161	0.084	0.011

*BP* blood pressure, *HDL* high-density lipoprotein, *LDL* low-density lipoprotein, *HOMA-IR* homeostasis model assessment-insulin resistance, *CRP* C-reactive protein

^a^Log-transformed variables for analysis. Simple linear regression used

Simple and multiple linear regression models for the associations of adiponectin with the domains of cognitive function are shown in Table 3. In crude models, adiponectin concentration was associated with memory, reaching statistical significance only for the delayed recall memory. In additional analyses, associations between adiponectin with BMI (β − 0.042, p = 0.200), C-reactive protein (β − 0.069, p = 0.037), and with HOMA-IR (β − 0.092, p = 0.005) were detected. In model 1 of multiple linear regression, adiponectin levels showed to be associated with delayed recall memory (Table 3), while prediabetes with learning memory (β − 0.080; p = 0.015) and delayed recall memory (β − 0.083; p = 0.012). After adjustments (model 2), adiponectin but not prediabetes maintained independently associated with delayed recall memory. In the same model, significant associations of delayed recall memory with the female sex [β 0.243 (95% CI 0.675; 1.212)], age [β − 0.110 (95% CI − 0.071; − 0.018)], and education level [β 0.205 (95% CI 0.535; 1.063)] were also detected. The inclusion of the interaction variable adiponectin*HOMA-IR did not change the association between adiponectin levels with improved delayed recall memory.

**Table 3:** Coefficients (95% confidence interval) of the association between domains of cognitive function and adiponectin levels using linear regression

	Crude model	p-value	Multiple linear regression			
			Model 1	p-value	Model 2	p-value
Learning memory	0.062 (− 0.012; 0.449)	0.063	0.063 (− 0.009; 0.451)	0.059	0.045 (− 0.068; 0.386)	0.170
Delayed recall memory	0.084 (0.033; 0.266)	0.012	0.085 (0.035; 0.267)	0.011	0.067 (0.006, 0.234)	0.040
Recognition memory	0.035 (− 0.025; 0.082)	0.298	–	–	–	–
Semantic verbal fluency test	− 0.011 (− 0.337; 0.242)	0.746	–	–	–	–
Phonemic verbal fluency test	− 0.006 (− 0.290; 0.238)	0.849	–	–	–	–
Trail making test	− 0.015 (− 5.153; 3.271)	0.661	–	–	–	–

Model 1: adjusted for the presence of prediabetes

Model 2: adjusted for sex (p < 0.001), age (p < 0.001), education (p < 0.001), physical activity, alcohol use, smoking, depression, thyroid dysfunction, BMI, HOMA-IR, hypertension, dyslipidemia, C-reactive protein

## Discussion

Our finding of association of memory domains with adiponectin levels at midlife suggests a role of this adipocytokine in early identification of high-risk individuals to cognition impairment, and supports previous assumption that it could represent a promising therapeutic target to protect against dementia. Independently of the recognized adverse effects of aging and abnormalities of glucose metabolism in the cognitive function, adjustments for both did not abolish the association of adiponectin with the delayed recall memory. Considering that insulin resistance is an underlying mechanism of type 2 DM and cognition impairment, and that adiponectin improves insulin signaling and glucose uptake, we suggest that the determination of this adipocytokine level could help to detect at-risk individuals before advanced age, in presymptomatic stages of the diseases.

The sample studied had some particularities, such as having included middle-aged highly-educated individuals and predominance of women. A neuroprotective role of schooling, able to elevate cognitive reserve, has been reported [32, 33], which could have contributed to optimal cognitive performance seen in participants of the present study. Our findings indicated better cognition scores in women compared to men. This finding is in agreement with studies investigating age- and sex-related cognitive performances, in which healthy men showed greater cognitive decline than women [34, 35], but differs from others [36]. Better scores observed among women from our study could be attributed in part to a better lifestyle than men and to their high education level. Actually, low education had been already associated with a fivefold increase in prevalence of cognition decline in a study conducted in Latin America [37]. We speculate that the ELSA highly-educated participants of both sexes might have a great cognitive reserve [32, 33], that could be protecting them against cognition impairment. In women, their good performance could be also explained by high proportion of premenopausal ones, in whom estrogen level could be preserving memory [38].

Based on a recognized tool to dementia assessment (CERAD), participants with mild disturbance of glucose tolerance (prediabetes) already showed a worse cognitive performance (namely learning and delayed recall memory) when compared to normotolerant ones. Association of dysglicemia and cognition dysfunction was previously described with predominance in male sex at high cardiovascular risk [39–41]. Although we had observed higher proportion of men with prediabetes in our study, sex-related difference cannot be assured since a convenience sample was studied. As expected, prediabetic participants were older and had worse insulin sensitivity and metabolic profile, which are known to impair cognitive function [12].

We verified that memory domains, but not fluency and trail tests, were worse in prediabetic than in normal tolerant individuals. Memory, which is mainly controlled by hippocampal neurons, has been shown as the first domain affected during the cognitive decline process [42]. Since glucose uptake by the hippocampus depends on insulin, several lines of investigation have implicated insulin resistance in the development of dementia [10, 12, 15] that manifests late in life. Our sample was composed of middle-aged individuals—in whom clinically relevant cognitive impairment is uncommon—but with a high frequency of prediabetes. Therefore, findings of our study suggest that the presence of mild disturbances of glucose metabolism, prior to overt DM, may deserve screening for cognition function independently of the age. Considering that an early detection of cognitive impairment in midlife could improve efficacy of preventive measures, risk assessment becomes even more important in prediabetes due to its potential to accelerate rate of cognitive decline [43, 44]. Few studies have been conducted in prediabetic individuals and have suggested better outcomes [39, 43, 45].

The most important finding of our study is related to the adiponectin, involved in the mechanism of neuronal injury, potentially able to improve risk detection of neurodegenerative diseases. Although still within reference ranges [46], higher adiponectin levels were associated with better delayed recall memory, independently of demographic variables and glucose metabolism disturbance. Since cerebral insulin resistance leads to increased amyloid β accumulation which causes synaptic damage [15], the insulin-sensitizing effect adiponectin seems desirable to preserve memory [47, 48]. Additionally, our final model of linear regression was consistent with the importance of aging for memory deterioration and education for memory maintenance [49]. Even controlling for these variables, adiponectin values, but not prediabetes, maintained significant association with memory. We interpreted that such association could be indicating an adiponectin-induced neuroprotective effect. It was demonstrated that stimulation of AdipoRs induces benefits not only on insulin signaling but also on inflammation and oxidative stress [50, 51]. In animals, when administered in the brain, adiponectin was able to suppress cytokines production by microglia [52]. Also, adequate adiponectin levels induced inhibition of TNF-α inflammatory effects and were able to prevent circulating inflammatory cytokines to cross the blood–brain barrier [53]. Therefore, its insulin-sensitizing, anti-inflammatory and antioxidant properties should be protecting against amyloid β accumulation and neuronal damage [54].

Taking adiponectin beneficial effects into consideration, novel therapies for Alzheimer’s disease, based on AdipoR agonists, have been proposed. However, such proposition contrasts with some studies conducted in elderly (age ≥ 70 years) that have described increased adiponectin levels in Alzheimer’s disease [55, 56]. The “adiponectin paradox” has been reported in association with a metabolically healthy obese phenotype. These findings reflect the complexity of therapy strategies addressed to adiponectin receptor signaling in aging-related chronic diseases [57].

We considered that the weak but significant association detected between memory and adiponectin in middle-aged individuals suggests that a lower adiponectin level could be indicative of an increased risk for cognitive impairment, when interventions might be more effective. Considering the growing proportion of older people for the next decades worldwide, earlier identification of individuals at higher risk is desirable in an attempt to delay dementia and preserve quality of life.

Our study has the limitation of having investigated a specific population at-risk for type 2 DM, that impedes extrapolation to the general population. However, it had the strength of having studied a large sample of middle-aged individuals with preserved neurocognitive function. Our results contribute to the knowledge about prevalent insulin resistance-linked diseases, in which adiponectin seems to play an important protective role at least during midlife. However, our cross-sectional analysis precludes investigating causality. The ELSA-Brasil cohort is promising to examine prospectively the ability of serum adiponectin to predict cognitive impairment in the middle-aged individuals at its baseline.

## Conclusions

The association of memory with adiponectin in middle-aged individuals, preceding overt diabetes, suggests that this adipokine might anticipate the cognitive function impairment when preventive strategies could be more effectively implemented. Whether adiponectin circulating levels could be useful to identify those at a higher risk for cognitive dysfunction before advanced age need to be prospectively investigated. Deepening knowledge about novel therapies against Alzheimer’s disease, based on the neuroprotective effect of adiponectin in amyloid B neurotoxicity, is also necessary.
